# Big Data and Clinicians: A Review on the State of the Science

**DOI:** 10.2196/medinform.2913

**Published:** 2014-01-17

**Authors:** Weiqi Wang, Eswar Krishnan

**Affiliations:** ^1^School of MedicineStanford UniversityPalo Alto, CAUnited States

**Keywords:** big data, database, medical informatics, clinical research, medicine

## Abstract

**Background:**

In the past few decades, medically related data collection saw a huge increase, referred to as big data. These huge datasets bring challenges in storage, processing, and analysis. In clinical medicine, big data is expected to play an important role in identifying causality of patient symptoms, in predicting hazards of disease incidence or reoccurrence, and in improving primary-care quality.

**Objective:**

The objective of this review was to provide an overview of the features of clinical big data, describe a few commonly employed computational algorithms, statistical methods, and software toolkits for data manipulation and analysis, and discuss the challenges and limitations in this realm.

**Methods:**

We conducted a literature review to identify studies on big data in medicine, especially clinical medicine. We used different combinations of keywords to search PubMed, Science Direct, Web of Knowledge, and Google Scholar for literature of interest from the past 10 years.

**Results:**

This paper reviewed studies that analyzed clinical big data and discussed issues related to storage and analysis of this type of data.

**Conclusions:**

Big data is becoming a common feature of biological and clinical studies. Researchers who use clinical big data face multiple challenges, and the data itself has limitations. It is imperative that methodologies for data analysis keep pace with our ability to collect and store data.

##  Introduction

Big data refers to very large datasets with complex structures that are difficult to process using traditional methods and tools. The term process includes, capture, storage, formatting, extraction, curation, integration, analysis, and visualization [1-9]. A popular definition of big data is the “3V” model proposed by Gartner [10], which attributes three fundamental features to big data: high volume of data mass, high velocity of data flow, and high variety of data types. The notion of big data can be traced back to the 1970s [11-13] when scientists realized that they lacked the tools to analyze datasets of large size. In those days, big data was merely several to hundreds of megabytes [14]; now datasets of terabytes are common [15, 16]. Therefore, the “big” in big data reflects the limits of data storage and computational power existing at a given point in time.

Table 1 shows the growth of global big data volume and computer science papers on big data since 2009. This table exemplifies that stored data will be in the tens of zettabytes range by 2020, and research on how to deal with big data will grow exponentially as well.

Big data is gathered in many disciplines and is made possible by ubiquitous information-sensing devices and software [19]. An example is web logs: websites such as Google or Facebook automatically record user information at each visit. Other examples come from the stock market [20], earthquake surveillance [21], political elections [22], behavioral studies [23], sports [24], pharmaceutical reports [25], health care [26, 27], electronic medical records [28], imaging data [29], genome data [30, 31], and entrepreneur transaction records [32]. Data collection is sometimes interdisciplinary. As an example, a sudden increase in Google search terms such as “flu symptoms” and “flu treatments” can be used to predict an increase in flu patients presenting to hospital emergency rooms [33]. This example also demonstrates that big data has promising predictive power and return on investment. Return on investment of big data has also been suggested for clinical big data [34, 35].

**Figure 1:**
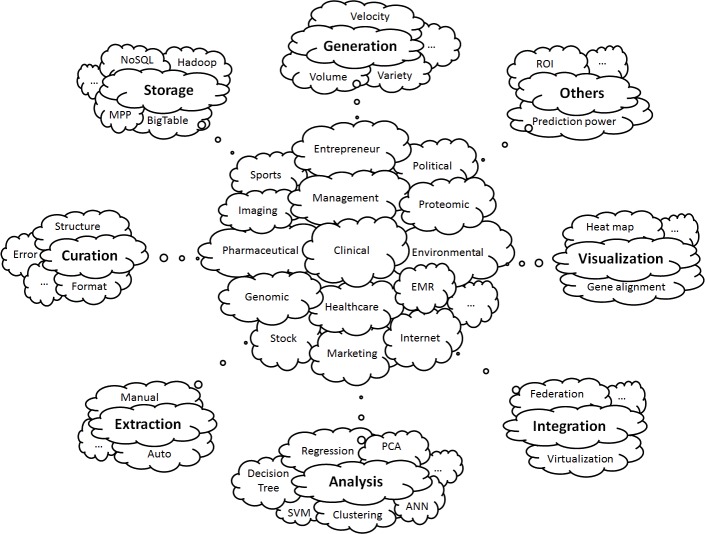
A schematic of the issues surrounding storage and use of big data. Clinical big data, as well as big data in other disciplines, have been surrounded by a number of issues and challenges, including (but not limited to): generation, storage, curation, extraction, integration, analysis, visualization, etc. ANN: artificial neuron network; EMR: electronic medical record; MPP: massively parallel-processing; PCA: principle component analysis; ROI: return of investment; SVM: support vector machine.

Although arguably valuable, big data is difficult to analyze due to the massive volume of the raw data and its diversity, as shown in Figure 1. Therefore, instead of the raw big data, a large dataset is often extracted from the raw data to generate a secondary storage of data for analysis purposes. This data extraction is applied, for example, when a computer tomography scan is involved in clinical trials and only the physician diagnosis based on the scan is included in data analysis. Similarly, a large volume of descriptive data on various kinds of samplings, tests, or assays can be extracted with only the key parameters kept. As a consequence, the data analyzed in clinical medicine is usually from secondary datasets that contain only data of interest. The secondary datasets, although still large, are not terabytes in size. Additionally, due to the nature of clinical trials, a large dataset in clinical medicine usually does not have an overwhelming number of samples. Kjaergard et al [36] defined clinical trials with 1000 or more participants as large, and the studies in clinical medicine titled big/large, data/dataset generally have thousands of attributes, but only hundreds of samples [37-39].

For this paper, we reviewed the literature to determine the features of clinical big data and determine the methods used for manipulation and analysis of these data. This paper is focused on clinical medicine rather than general health care issues; therefore, we mainly reviewed the studies that appeared relevant to clinicians. We examined the selected studies to extract information on research interests, goals, and achievements, and the implemented methodologies. Our intention was not to conduct an exhaustive systematic review, but instead to enable a literature-based discussion of how the big data issue has been addressed in clinical medicine. Based on our findings, we discuss the challenges and limitations of analysis of large clinical datasets.

**Table 1:** Global growth of big data and computer science papers on big data.

Year	Data volume, ZB^a,c^	Conference papers, CS^b,c^	Journal papers, CS^c^
2009	1.5	12	7
2010	2	26	7
2011	2.5	32	23
2012	3	78	47
2015	8	?	?
2020	44	??	??

^a^Data from *oracle* [17].

^b^Data from *Research Trends* [18].

^c^CS, computer science; ZB, zettabytes (1 zettabyte = 1000 terabytes = 10^6^ petabytes = 10^18^ gigabytes, GB).

##  Methods

We conducted a literature review to identify studies on big data in medicine, especially clinical medicine. We used different combinations of keywords to search PubMed, Science Direct, Web of Knowledge, and Google Scholar for literature of interest, mainly from the last 10 years. The key words were: "big data medicine", "large dataset medicine", “clinical big data”, “clinical large dataset”, “clinical data warehouse”, “clinical database”, “clinical data mining”, “biomedical big data”, “biomedical database”, “biomedical data warehouse”, “healthcare big data”, “healthcare database”, and “healthcare data warehouse”.

## Results

### Big Data in Clinical Medicine

Big data plays an important role in medical and clinical research and has been leveraged in clinically relevant studies. Major research institute centers and funding agencies have made large investments in the arena. For example, the National Institutes of Health recently committed US $100 million for the big data to Knowledge (BD2K) initiative [40]. The BD2K defines “biomedical” big data as large datasets generated by research groups or individual investigators and as large datasets generated by aggregation of smaller datasets. The most well-known examples of medical big data are databases maintained by the Medicare and Healthcare Cost and Utilization Project (with over 100 million observations). One of the differences between medical big data and large datasets from other disciplines is that clinical big data are often collected based on protocols (ie, fixed forms) and therefore are relatively structured, partially due to the extraction process that simplify raw data as mentioned above. This feature can be traced back to the Framingham Heart Study [41], which has followed a cohort in the town of Framingham, Massachusetts since 1948. Vast amounts of data have been collected through the Framingham Heart Study, and the analysis has informed our understanding of heart diseases, including the effects of diet, exercise, medications, and obesity on risk [42]. There are many other clinical databases with different scopes, including but not limited to, prevalence and trend studies, risk factor studies, and genotype-phenotype studies.

### Prevalence and Trend Studies

One of the major uses for clinical big data is in analysis of the prevalence or trends of a disease or phenotype among different populations. An early big data study evaluated a cohort consisting of 890,394 US veterans with diabetes followed from 2002 through 2006 [43]. Bermejo-Sanchez et al [44] observed 326 of the birth defect Amelia among 23 million live births, stillbirths, and fetal anomalies from 23 countries and 4 continents, and found the trend of higher prevalence of Amelia among younger mothers. Histological features that differ between chronic idiopathic inflammatory bowel disease and normality and between Crohn’s disease and ulcerative colitis were identified in 809 large bowel endoscopic biopsies [45]. Kelly et al [46] studied the prevalence of hip abnormalities of 8192 subjects with hemophilia A and B. Siregar et al [47] performed a population-based study on patients after cardiac surgery in all 16 cardiothoracic surgery centers in the Netherlands. Elshazly et al [48] examined 1.3 million US adults for patient-level discordance of non-high-density lipoprotein cholesterol and low-density lipoprotein cholesterol. Chan and McGarey [49] summarize how large datasets can be analyzed to achieve population-based conclusions, specifically for determination of secular trends, health disparities, geographic variation, and evaluation of specific diseases and treatments. This paper also summarized the strengths and limitations of large-sized datasets and addressed issues such as missing data and bias. These issues will also be discussed in brief below.

### Risk Factor Studies

Clinical big data can also be used to determine causality, effect, or association between risk factors and the disease of interest. Ursum et al [50] examined the relationships between seroconversion and patient age with inflammatory effects of autoantibodies in 18,658 rheumatoid arthritis patients and controls, and showed that citrullinated proteins and peptides were more reliable markers for rheumatoid arthritis than was Immunoglobulin M rheumatoid factor. Ajdacic-Gross et al [51] examined the data on 11,905 Swiss conscripts from 2003 for stuttering and found that there was no single overwhelming risk factor for stuttering, although premature birth and parental alcohol abuse appeared influential. Data collected on 14,433 patients from the 155 Veterans Administration medical centers in all 50 US states, Puerto Rico, and the District of Columbia were used to identify the alcohol dependence of medications [52]. By analysis of 53,177 cases of contrast administration in 35,922 patients from the Radiology and Cardiac Catheterization Laboratory databases, an increase in contrast nephropathy was associated with use of sodium bicarbonate [53]. Echocardiography and electrocardiogram-gated single-photon emission computed tomography traces for the evaluation of left ventricular ejection fraction were compared in 534 patients [54]. Zhang et al [55] examined clinical data of 16,135 adult patients and elucidated the relationships between glycemic, blood glucose level, and intake of insulin with mortality. Mitchel et al [56] studied the effect of 2 types of insulin on 7720 patients selected from 8 million in UK. Kobayashi et al [57] analyzed 19,070 records on right hemicolectomy from 3500 Japanese hospitals and successfully developed a risk model. It should be noted that in these studies, the terms of “association” and “causality” must be rigorously distinguished; most of the studies claimed association, whereas causality was rarely asserted.

### Genotype–Phenotype Studies

With the advancement of genotyping technology, an increasing amount of risk-factor studies have attempted to assess association on the genetic level through evaluation of gene expression and/or genomic data obtained from patients and controls. For example, clinical and genetic data from 5700 patients who had been treated with warfarin were used to create an algorithm to estimate the appropriate dose [58]. Causality of autism spectrum disorders has been investigated by analysis of 31,516 clinical cases on copy number variation in patients versus 13,696 controls [59]. Koefoed et al [60] made efforts to assess the effects of signal transmission and calculated all combinations of three genotypes from 803 single-nucleotide polymorphism (SNP) genotypes (2.3 billion combinations) for 1355 controls and 607 patients with bipolar disorder. These studies are similar to risk-factor studies, yet often the big data is significantly larger in volume due in genetic analyses than in risk-factor studies.

### Method Development Studies

A number of studies have taken advantage of clinical big data to establish new methods or techniques, or to develop new tools to enable analysis of data and decision making. In a typical example, Hill et al [61] designed an interface to use clinical data to evaluate risk ratios for various diseases to aid in evaluation of treatment options. Liu et al [62, 63] have used large-scale data analysis to optimize diagnosis of breast cancer from full-field digital mammography images. Lin et al [64] made efforts to formalize the phenotype variable in the database Genotypes and Phenotypes. Stephen et al [65] developed an algorithm to categorize pediatric patients presenting with respiratory distress into different subtypes using clinical variables from a clinical data warehouse. Clinical data warehouses or databases have been created from radiotherapy clinical trial data [66], gene mutations [67], cancer patient data [68, 69], kidney disease patient data [70], and gastrointestinal surgery patient data [71]. Additionally, studies have focused on personalized big data [72], citizen-centric health care versus patient-centric health care [72, 73], medication orders [74, 75], and decision making and information management/retrieval in general [75-80]. The dramatic increase in the number of studies with large scope in the past few years indicates an increasing desire of researchers to manipulate clinical big data; “big data-assisted clinics” may be expected in the near future.

## Discussion

### Diversity of Data in Clinical Medicine

The huge body of medical research that has been performed using large datasets demonstrates the broad spectrum of data resources used and shows that the structure of the medical dataset depends on the research question. Data from different subareas of medical research have broad diversity in terms of numbers of entries, types of data stored (or levels), dimensionality, and sample size [81]. Datasets obviously differ greatly in size: gene expression datasets derived from high-throughput microarray and next-generation sequencing technologies, such as those that analyze SNPs and copy number variations, tend to be massive, whereas clinical trial dataset are not as big. Phan et al [82] suggested that data in medicine be divided into four different levels: the molecular level (eg, genome data), cellular and tissue level (eg, stem cell differentiation data), clinical and patient level (eg, clinical trial data), and biomedical knowledge base level (ie, a comprehensive data collection). Additionally, data tend to have different levels of dimensionality (ie, number of attributes or parameters, *p*) and sample sizes (ie, number of records/entries, n). Typical datasets fall into one of three categories, as summarized by Sinha et al [83]: large n, small *p*; small n, large *p*; and large n, large *p*. Thanks to advancements in computational technology, most algorithms can handle low-dimensional data (ie, large n, small *p*) without encountering significant difficulty.

Most clinical data, however, is high-dimensional (ie, small n, large *p* or large n, large *p*) due to a limited number of patients. One typical example comes from a study of 69 Broca’s aphasic patients (ie, n=69) who were tested with nearly 6000 stimulus sentences (ie, *p*~6000) [84]. With similar dimensionality, Mitchell et al [39] studied bipolar disorder where the sample consisted of only 217 patients. For high-dimensional data, each point, sample, or element is described by many attributes [83] with the involvement of the “curse of dimensionality” [85]. Because high-dimensional data are sparse in dimensions, most classification or clustering approaches do not work well because the increase in problem space reduces the overall density of data samples. To solve this problem, compression methods and significance testing are usually used to either reduce the dimensionality or select relevant features before data analysis by some sort of data preprocessing [83].

### Methods for Manipulation of Clinical Big Data

####  Technologies for Data Storage and Handling

Due to the massiveness and complexity of big data, nonrelational and distributed databases such as Apache Hadoop [86], Google BigTable [87], NoSQL [88], and massively parallel-processing databases are used rather than traditional relational databases to store data. A large number of biostatistics software packages have been used to handle large clinical datasets, some of which enabled the features of cloud-based or distributed computing. Popular software packages include, but are not limited to, SAS [36, 51-53], Mplus [51], SPSS [36, 39, 45], PP-VLAM [89], Stata [90], and R [91]. These technologies and tools greatly facilitate the handling of big data.

#### Methodologies for Data Preprocessing

Clinical raw big data can be highly diverse and uninformative without preprocessing. Extraction of a diagnosis from raw computer tomography data is an example of one of the predominant manners in which clinical big data are preprocessed. This type of processes relies on a specialist’s personal expertise and can be a source of bias. Most early analyses of big data, including that collected by the Framingham Heart Study adopted some form of preprocessing; therefore, challenges exist in curation [6]. As an alternative to expert preprocessing, computational algorithms or statistical approaches, including compression methods, significance testing, or normalization [92] can be implemented to preprocess raw big data. This methodology may also introduce bias and can cause uncertainty problems during data integration.

In some scenarios, visualization can be a part of data preprocessing (as well as result exhibition). Typical examples in this regard include the use of heat maps [93], gene alignments [94], protein structure visualization [95], scatterplot matrix, tree visualization, network visualization, parallel coordinates, stacked graphs, etc. When the big data of interest are scattered or stored at different resources, data integration [96, 97] and federation [98] is an important phase during data preprocessing. Approaches such as the Information Manifold [97], which allows browsing and querying of multiple networked information sources, can provide solutions to uncertainty problems after data integration and mapping [99].

#### Statistical Approaches to Data Analysis

A number of popular statistical methods have been implemented in clinical data analysis. The most common include linear regression and logistic regression [30], latent class analysis [100], principle component analysis [101], and classification and regression trees [100]. Additionally, logarithmic and square-root transformations [58], naive Bayes methods [102], decision trees [103], neural networks [104], support vector machines [105], and hidden Markov models [83] are also used to study problems in medical data.

When a dataset is not overly complicated, a single test (eg, a simple Student’s *t* test) should be powerful enough to reject a null hypothesis, and single hypothesis testing is the methodology to adopt [106]. Sometimes one cannot establish the significance of a hypothesis until different statistical tests have been applied to the same dataset. Multiple testing is often used to identify correlations that deserve further investigation [107]. Algorithms for false discovery rate [108] and family-wise error rate [109] calculation have been implemented for multiple testing in studies on gene expression data and datasets with similar levels of complexity.

### Challenges and Limitations of Use of Clinical Big Data

#### Overview

Big data itself has many limitations. These limitations include “adequacy, accuracy, completeness, nature of the reporting sources, and other measures of the quality of the data”, as summarized previously [110]. The consequences of these limitations are succinctly summarized in the book titled “Models. Behaving. Badly.” [111]. Modeling can often lead to a biased statistical correlation or inference, sometimes known as a “false discovery”. Clinical big data users face a large spectrum of challenges, including but not limited to sample size, selection bias, interpretation problem, missing values, dependence problems, and data handling methodologies.

#### Sample Size

One of the counterintuitive challenges in analysis of big data clinical datasets is that sometimes the sample size is not as big, compared with the number of attributes to allow statistically significant analysis. Population survey methods are sometimes adopted because these methods can provide larger datasets. However, the authenticity and accuracy of this type of data are arguably limited; hence, survey methods cannot be reliably used to produce an adequate description or prediction [39].

#### Selection Bias

Any dataset is a selection of data rather than the whole data world; therefore, selection bias is a very real limitation [112] even if the sample size is big. In that sense, all studies of clinical data have this limitation to some degree [39].

#### Interpretation Problem

Gebregziabher et al [43] stated that the datasets generated through many translational research projects to answer questions of public health interest are not self-explanatory due to complexity and inadequate description/documentation of the dataset's parameters and associated metadata. The methodologies for interpreting the data can therefore be subject to all sorts of philosophical debate. For example, the data may not be totally naïve or objective and interpretation may be biased by subjective assumptions and/or manipulations by individual analysts.

#### Missing Values

It is common problem that large datasets have missing values, and in many cases the problem can be significant [44]. A typical example is the Framingham Heart Study where data on serum uric acid are largely missing. Additionally, the covariates (ie, attributes) may not fully capture the degree of risk for patients and may cause uncertainty in results [53].

#### Dependence Problems

One issue that has been often neglected is the dependence of data. Dependence between either attributes or samples in datasets can cause the degrees of freedom to decrease and/or some statistical principles to no longer apply. Examples of this are found when the same patients are evaluated multiple times through follow-up and when correlations in gene expression are drawn based on samples from different patients treated with similar medications [83]. As many statistical methods do not account for dependence, results from these tests may be unreliable if this issue is not properly addressed before the data analysis.

####  Data Handling Methodologies

Effective processing of big data has always been a challenge. One must consider all the aspects of the dataset, including collection, curation, extraction, integration, interpretation, imputation, and selection of appropriate statistical methods, during processing and analysis. It has been claimed that analyses of large datasets are often suboptimal due to the researcher’s lack of knowledge of the available tools and methodologies [83]. On the other hand, algorithms to handle big data are also underdeveloped to some extent and deserve more attention [113].

###  Conclusions

This paper reviewed studies that analyzed clinical big data and that discuss issues related to data storage and analysis. Big data is becoming a common feature of biological and clinical studies. Today, a single biophysical researcher can generate terabytes of data in hours. Over the last decade, clinical datasets have grown incredibly in size, mostly due to use of modern technologies for collection and recording of data. Researchers who use clinical big data face multiple challenges, and the data itself has limitations. It is imperative that methodologies for data analysis keep pace with our ability to collect and store data.

## Abbreviations

BD2K: Big Data to Knowledge
CS: computer science
SNP: single-nucleotide polymorphism
ZB: zettabytes
